# Hot Water in Therapeutics

**Published:** 1883-06

**Authors:** 


### Hot Water in Therapeutics.

Several years ago I learned in my personal experience that no agent relieves nausea and vomiting so satisfactorily and promptly as water as hot as can be drunk. Since then, I have used it in a large number of cases, and it has been uniformly reliable. The following classification may be made of the cases in which it has been used:

(1.) Cases in which nausea and vomiting occurred at the onset or during the course of acute febrile disease.

(2.) Cases in which these symptoms were caused by overloading the stomach when its functions had been impaired by protracted disease.

(3.) Cases in which they were produced by nauseous medicines (not emetics) at the time they were taken.

(4.) Cases of acute gastritis caused by the ingestion of irritants.

(5.) Cases in which these symytoms were purely reflex.

(6.) Cases of chronic gastritis.

(7.) Cases of colic in newly born infants.

(8.) Cases of flatulent distention of the stomach.

Among the cases of Class 1 was a case of diphtheria and one of puerperal septicæmia, as well as one of tuberculosis, in which the stress of the disease fell upon the digestive apparatus. In each of these a half glass of hot water always gave prompt relief when every other remedy failed. The most impressive and permanent results of this remedy seem to be in cholera infantum—hot water being retained when everything else was rejected; and it would so compose the stomach that food could be given almost immediately afterward.

The nauseant medicines mentioned in Class 3 are often retained if given in hot water as a vehicle. When an enormous quantity of whisky has been drunk, and the stomach will not tolerate anything else, hot water will be retained, and then food can be given.

Hot water is less satisfactory in vomiting of pregnancy, yet it is of considerable value in many cases.

In the various manifestations of indigestion, classed 6, 7 and 8—hot water is almost invariably followed by good results. In dyspepsia it may be given before each meal, as well as at other times, to cause the discharge of any undue amount of gas in the stomach by eructation. In this way it affords relief to young infants suffering from colic, and it is rarely necessary to prescribe anything else. It has been used successfully in a case of severe palpitation of the heart from dyspepsia.

The *decongestive* and hæmostatic action of hot water have been variously accounted for by gynæcologists. Dr. Pitcher, of Detroit, thought that when applied to a bleeding vessel, the immediate effect is dilatation, which sufficiently slows the current to form a clot; and constriction occurring afterward, the clot is firmly held and the lumen of the vessel closed.

Dr. Emmet says that the direct result is relaxation of the coat and vascular turgescence; afterward, if continued, reaction follows and contraction occurs.

Carl Ritcher, of Berlin, thinks "the contact of the hot water with the partially denuded inner wall of the uterus causes a slight inflammatory irritation, an œdematous transudation, and a swelling of the tissues, principally the submucous, intermuscular, and perivascular connective tissue, by which the blood vessels become compressed and their lumina thereby occluded."

The action of hot water upon the uterine or gastric mucous membrane or upon abnormally full or bleeding vessels in any part of the body, may be readily and simply explained by a well-known physiological principle, viz.: That of watching a frog's foot while a needle is drawn across without injuring the membrane. The vessels will presently contract and close, and after remaining so for a few minutes, will dilate to respond no more, or only partially, to such stimulus. With a stronger stimulus, as that of gentle heat, they will again contract, and such contraction may be lost a day or two.

Wharton Jones found that cold causes speedy constriction, quickly followed by dilatation.

Beaumont, in his observations on St. Martin, found that the ingestion of cold water was followed by blanching of the gastric mucous membrane, quickly followed by more than normal redness. Now, from whatever cause nausea and vomiting may arise—from the direct contact of an irritant, or the effect of an emetic, or from reflex nervous influences, it is certain in many instances, and probable in all others, that the vaso-motor centers controlling the gastric blood supply are also influenced and gastric hyperæmia produced; and this condition being the link in the casual chain which is broken by the contact of the hot water on the gastric lining, the effect fails to follow. In flatulent distention of the stomach the muscular coat, impeded by the gaseous pressure, is excited to extraordinary work, and the gas is expelled. Patients who begin taking hot water to allay nausea, can not only take large quantities without inconvenience, but get to liking it; and at times, when little or no water can be taken, by drinking it hot, enough can be retained to fully meet the requirements of the organism. This fact has an important bearing in therapeutics. Often in both acute and chronic diseases, the issue depends solely on the amount of work that the kidneys will do. In many diseases, the structure of these organs, though not primarily affected by the morbid process, is liable to damage secondarily. The injury may be due rather to the concentration of the urine—the small amount of water—than to the absolute amount of solids; so that the kidneys become clogged with destructive, effete matter if sufficient water fails to flow through them. But dilution of the urine is not the only good resulting from the free drinking of water. The skin is put to work and carries off a large portion of the effete matter that would otherwise have to pass through the kidneys.—*Dr. Douglas Morton, in Louisville Medical News.*

